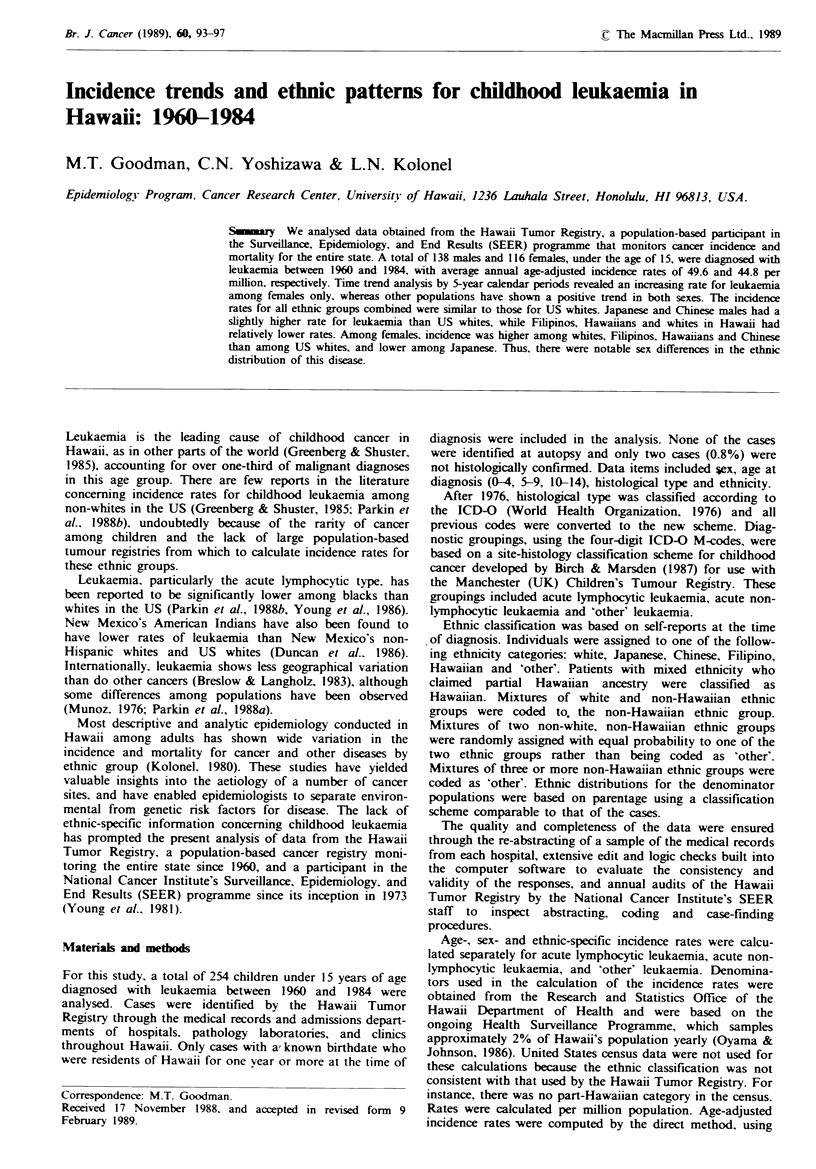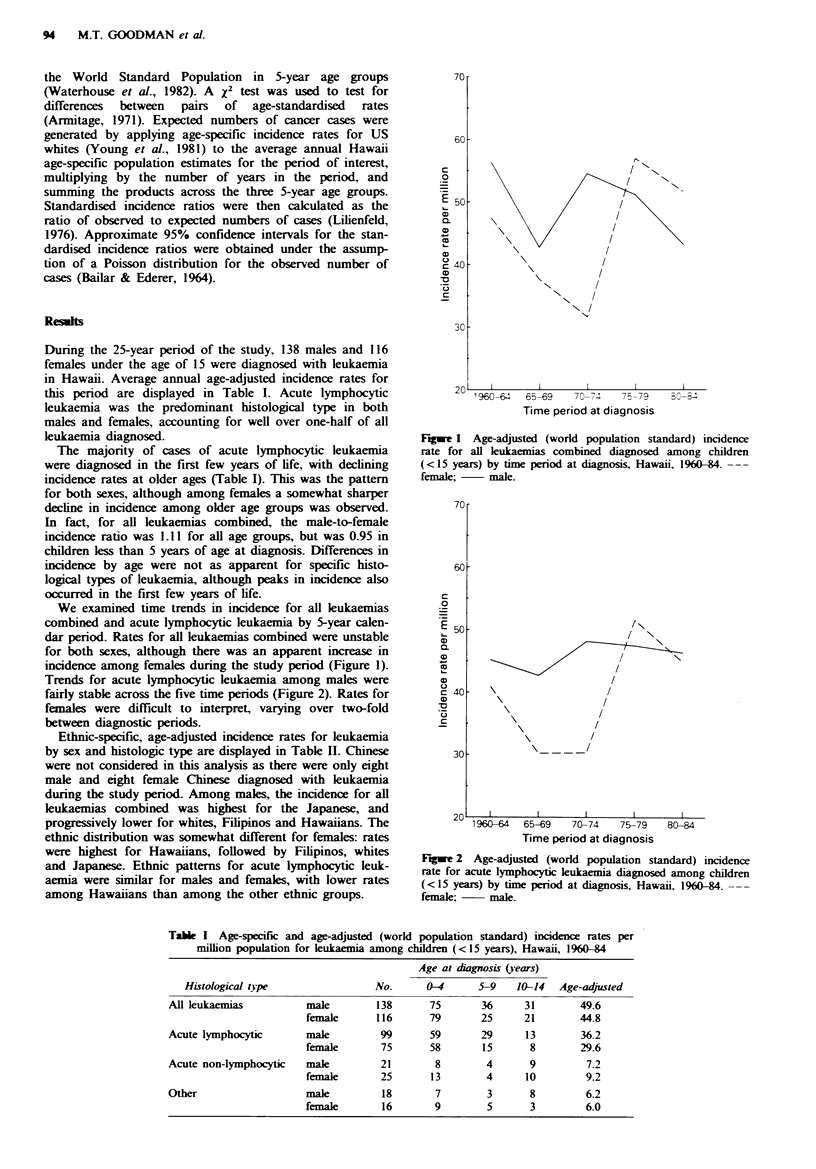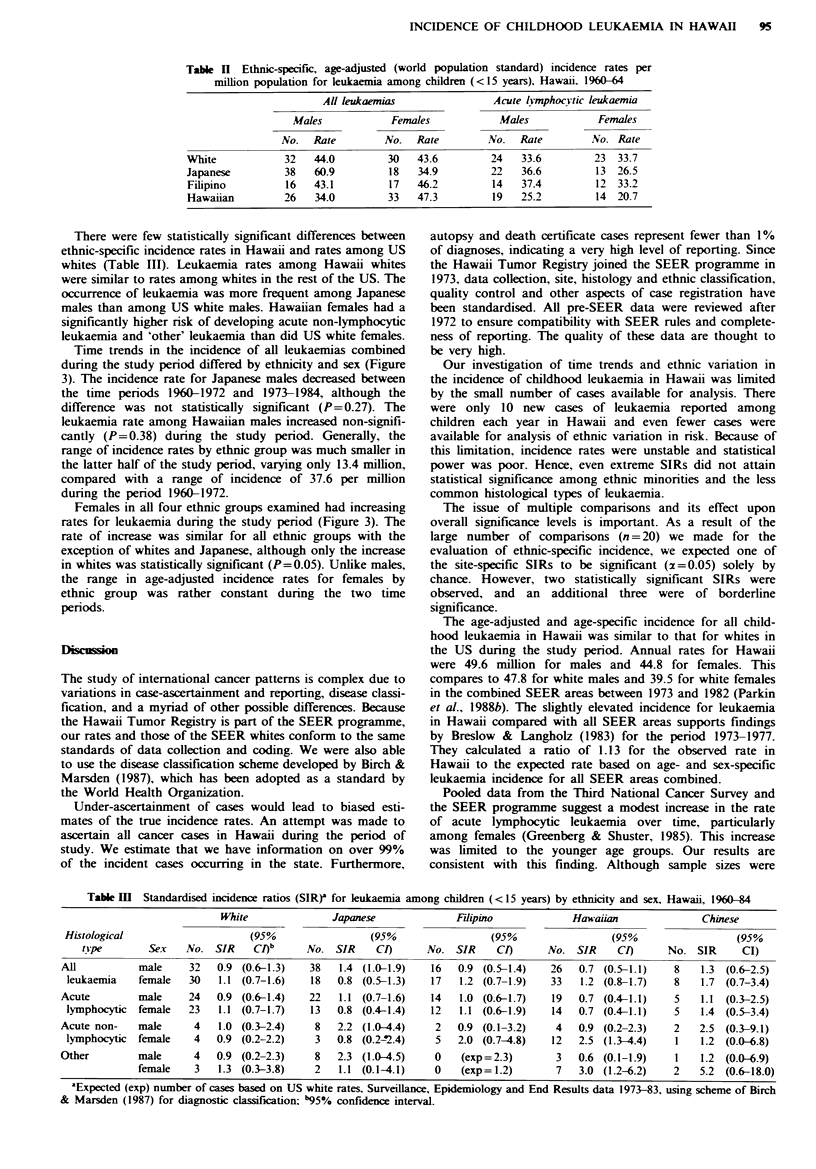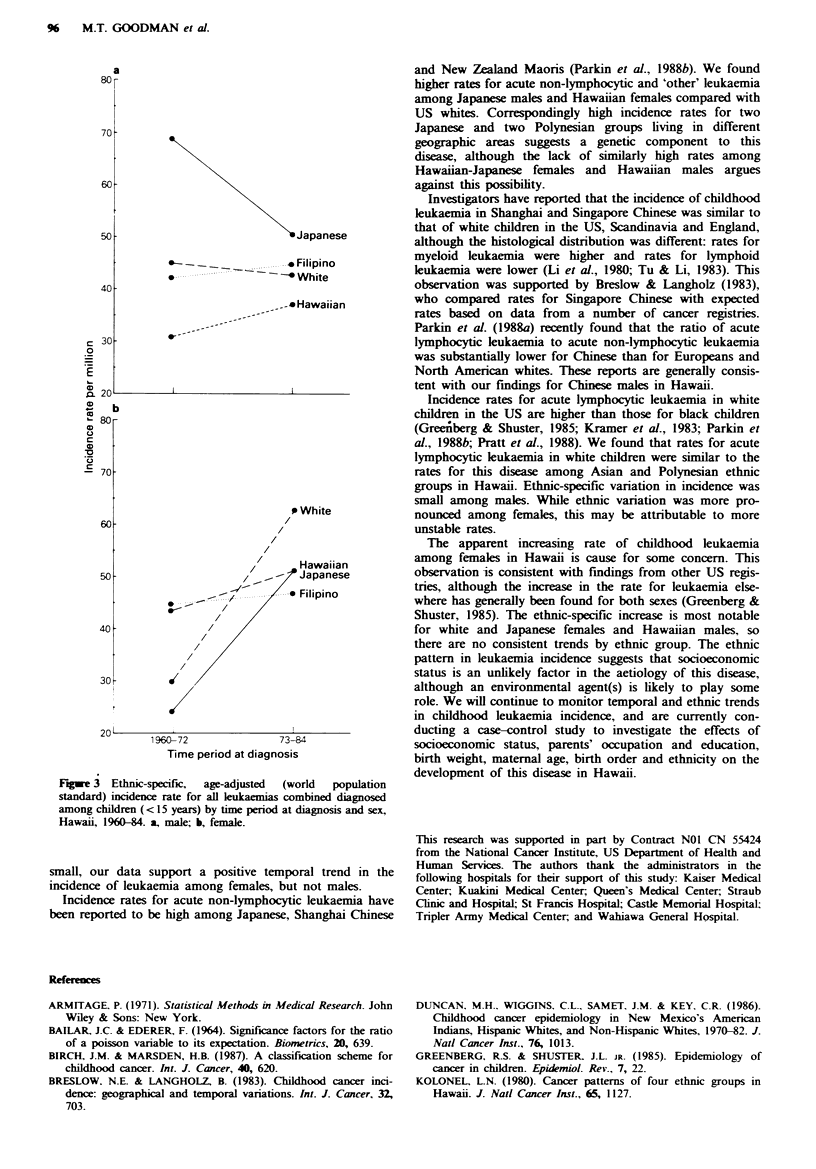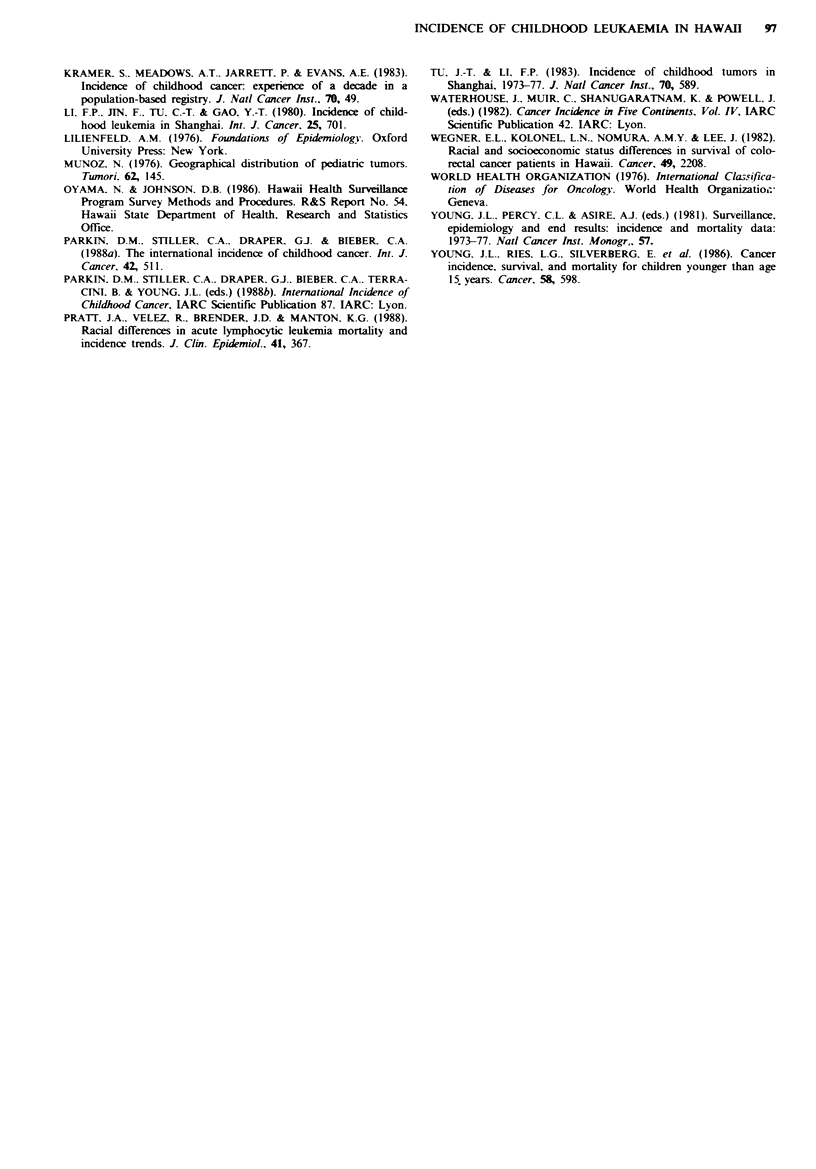# Incidence trends and ethnic patterns for childhood leukaemia in Hawaii: 1960-1984.

**DOI:** 10.1038/bjc.1989.227

**Published:** 1989-07

**Authors:** M. T. Goodman, C. N. Yoshizawa, L. N. Kolonel

**Affiliations:** Cancer Research Center, University of Hawaii, Honolulu 96813.

## Abstract

We analysed data obtained from the Hawaii Tumor Registry, a population-based participant in the Surveillance, Epidemiology, and End Results (SEER) programme that monitors cancer incidence and mortality for the entire state. A total of 138 males and 116 females, under the age of 15, were diagnosed with leukaemia between 1960 and 1984, with average annual age-adjusted incidence rates of 49.6 and 44.8 per million, respectively. Time trend analysis by 5-year calendar periods revealed an increasing rate for leukaemia among females only, whereas other populations have shown a positive trend in both sexes. The incidence rates for all ethnic groups combined were similar to those for US whites. Japanese and Chinese males had a slightly higher rate for leukaemia than US whites, while Filipinos, Hawaiians and whites in Hawaii had relatively lower rates. Among females, incidence was higher among whites, Filipinos, Hawaiians and Chinese than among US whites, and lower among Japanese. Thus, there were notable sex differences in the ethnic distribution of this disease.


Br. J. Cancer (1989), 60, 93-97 © The Macmillan Press Ltd., 1989

### Incidence trends and ethnic patterns for childhood leukaemia in Hawaii: 1960–1984

M.T. Goodman, C.N. Yoshizawa & L.N. Kolonel

*Epidemiology Program, Cancer Research Center, University of Hawaii, 1236 Lauhala Street, Honolulu, HI 96813, USA.*

**Summary** We analysed data obtained from the Hawaii Tumor Registry, a population-based participant in the Surveillance, Epidemiology, and End Results (SEER) programme that monitors cancer incidence and mortality for the entire state. A total of 138 males and 116 females, under the age of 15, were diagnosed with leukaemia between 1960 and 1984, with average annual age-adjusted incidence rates of 49.6 and 44.8 per million, respectively. Time trend analysis by 5-year calendar periods revealed an increasing rate for leukaemia among females only, whereas other populations have shown a positive trend in both sexes. The incidence rates for all ethnic groups combined were similar to those for US whites. Japanese and Chinese males had a slightly higher rate for leukaemia than US whites, while Filipinos, Hawaiians and whites in Hawaii had relatively lower rates. Among females, incidence was higher among whites, Filipinos, Hawaiians and Chinese than among US whites, and lower among Japanese. Thus, there were notable sex differences in the ethnic distribution of this disease.

Leukaemia is the leading cause of childhood cancer in Hawaii, as in other parts of the world (Greenberg & Shuster, 1985), accounting for over one-third of malignant diagnoses in this age group. There are few reports in the literature concerning incidence rates for childhood leukaemia among non-whites in the US (Greenberg & Shuster, 1985; Parkin *et al.*, 1988*b*), undoubtedly because of the rarity of cancer among children and the lack of large population-based tumour registries from which to calculate incidence rates for these ethnic groups.

Leukaemia, particularly the acute lymphocytic type, has been reported to be significantly lower among blacks than whites in the US (Parkin *et al.*, 1988*b*, Young *et al.*, 1986). New Mexico's American Indians have also been found to have lower rates of leukaemia than New Mexico's non-Hispanic whites and US whites (Duncan *et al.*, 1986). Internationally, leukaemia shows less geographical variation than do other cancers (Breslow & Langholz, 1983), although some differences among populations have been observed (Munoz, 1976; Parkin *et al.*, 1988*a*).

Most descriptive and analytic epidemiology conducted in Hawaii among adults has shown wide variation in the incidence and mortality for cancer and other diseases by ethnic group (Kolonel, 1980). These studies have yielded valuable insights into the aetiology of a number of cancer sites, and have enabled epidemiologists to separate environmental from genetic risk factors for disease. The lack of ethnic-specific information concerning childhood leukaemia has prompted the present analysis of data from the Hawaii Tumor Registry, a population-based cancer registry monitoring the entire state since 1960, and a participant in the National Cancer Institute's Surveillance, Epidemiology, and End Results (SEER) programme since its inception in 1973 (Young *et al.*, 1981).

### Materials and methods

For this study, a total of 254 children under 15 years of age diagnosed with leukaemia between 1960 and 1984 were analysed. Cases were identified by the Hawaii Tumor Registry through the medical records and admissions departments of hospitals, pathology laboratories, and clinics throughout Hawaii. Only cases with a known birthdate who were residents of Hawaii for one year or more at the time of diagnosis were included in the analysis. None of the cases were identified at autopsy and only two cases (0.8%) were not histologically confirmed. Data items included sex, age at diagnosis (0–4, 5–9, 10–14), histological type and ethnicity.

After 1976, histological type was classified according to the ICD-O (World Health Organization, 1976) and all previous codes were converted to the new scheme. Diagnostic groupings, using the four-digit ICD-O M-codes, were based on a site-histology classification scheme for childhood cancer developed by Birch & Marsden (1987) for use with the Manchester (UK) Children's Tumour Registry. These groupings included acute lymphocytic leukaemia, acute non-lymphocytic leukaemia and 'other' leukaemia.

Ethnic classification was based on self-reports at the time of diagnosis. Individuals were assigned to one of the following ethnicity categories: white, Japanese, Chinese, Filipino, Hawaiian and 'other'. Patients with mixed ethnicity who claimed partial Hawaiian ancestry were classified as Hawaiian. Mixtures of white and non-Hawaiian ethnic groups were coded to the non-Hawaiian ethnic group. Mixtures of two non-white, non-Hawaiian ethnic groups were randomly assigned with equal probability to one of the two ethnic groups rather than being coded as 'other'. Mixtures of three or more non-Hawaiian ethnic groups were coded as 'other'. Ethnic distributions for the denominator populations were based on parentage using a classification scheme comparable to that of the cases.

The quality and completeness of the data were ensured through the re-abstracting of a sample of the medical records from each hospital, extensive edit and logic checks built into the computer software to evaluate the consistency and validity of the responses, and annual audits of the Hawaii Tumor Registry by the National Cancer Institute's SEER staff to inspect abstracting, coding and case-finding procedures.

Age-, sex- and ethnic-specific incidence rates were calculated separately for acute lymphocytic leukaemia, acute non-lymphocytic leukaemia, and 'other' leukaemia. Denominators used in the calculation of the incidence rates were obtained from the Research and Statistics Office of the Hawaii Department of Health and were based on the ongoing Health Surveillance Programme, which samples approximately 2% of Hawaii's population yearly (Oyama & Johnson, 1986). United States census data were not used for these calculations because the ethnic classification was not consistent with that used by the Hawaii Tumor Registry. For instance, there was no part-Hawaiian category in the census. Rates were calculated per million population. Age-adjusted incidence rates were computed by the direct method, using

Correspondence: M.T. Goodman.
Received 17 November 1988, and accepted in revised form 9 February 1989.

the World Standard Population in 5-year age groups (Waterhouse et al., 1982). A $\chi^2$ test was used to test for differences between pairs of age-standardised rates (Armitage, 1971). Expected numbers of cancer cases were generated by applying age-specific incidence rates for US whites (Young et al., 1981) to the average annual Hawaii age-specific population estimates for the period of interest, multiplying by the number of years in the period, and summing the products across the three 5-year age groups. Standardised incidence ratios were then calculated as the ratio of observed to expected numbers of cases (Lilienfeld, 1976). Approximate 95% confidence intervals for the standardised incidence ratios were obtained under the assumption of a Poisson distribution for the observed number of cases (Bailar & Ederer, 1964).

### Results

During the 25-year period of the study, 138 males and 116 females under the age of 15 were diagnosed with leukaemia in Hawaii. Average annual age-adjusted incidence rates for this period are displayed in Table I. Acute lymphocytic leukaemia was the predominant histological type in both males and females, accounting for well over one-half of all leukaemia diagnosed.

The majority of cases of acute lymphocytic leukaemia were diagnosed in the first few years of life, with declining incidence rates at older ages (Table I). This was the pattern for both sexes, although among females a somewhat sharper decline in incidence among older age groups was observed. In fact, for all leukaemias combined, the male-to-female incidence ratio was 1.11 for all age groups, but was 0.95 in children less than 5 years of age at diagnosis. Differences in incidence by age were not as apparent for specific histological types of leukaemia, although peaks in incidence also occurred in the first few years of life.

We examined time trends in incidence for all leukaemias combined and acute lymphocytic leukaemia by 5-year calendar period. Rates for all leukaemias combined were unstable for both sexes, although there was an apparent increase in incidence among females during the study period (Figure 1). Trends for acute lymphocytic leukaemia among males were fairly stable across the five time periods (Figure 2). Rates for females were difficult to interpret, varying over two-fold between diagnostic periods.

Ethnic-specific, age-adjusted incidence rates for leukaemia by sex and histologic type are displayed in Table II. Chinese were not considered in this analysis as there were only eight male and eight female Chinese diagnosed with leukaemia during the study period. Among males, the incidence for all leukaemias combined was highest for the Japanese, and progressively lower for whites, Filipinos and Hawaiians. The ethnic distribution was somewhat different for females: rates were highest for Hawaiians, followed by Filipinos, whites and Japanese. Ethnic patterns for acute lymphocytic leukaemia were similar for males and females, with lower rates among Hawaiians than among the other ethnic groups.

**Figure 1** Age-adjusted (world population standard) incidence rate for all leukaemias combined diagnosed among children (<15 years) by time period at diagnosis, Hawaii, 1960–84. --- female; —— male.

**Figure 2** Age-adjusted (world population standard) incidence rate for acute lymphocytic leukaemia diagnosed among children (<15 years) by time period at diagnosis, Hawaii, 1960–84. --- female; —— male.

**Table I** Age-specific and age-adjusted (world population standard) incidence rates per million population for leukaemia among children (<15 years), Hawaii, 1960–84

| | | | *Age at diagnosis (years)* | | | |
|---|---|---|---|---|---|---|
| *Histological type* | | *No.* | *0–4* | *5–9* | *10–14* | *Age-adjusted* |
| All leukaemias | male | 138 | 75 | 36 | 31 | 49.6 |
| | female | 116 | 79 | 25 | 21 | 44.8 |
| Acute lymphocytic | male | 99 | 59 | 29 | 13 | 36.2 |
| | female | 75 | 58 | 15 | 8 | 29.6 |
| Acute non-lymphocytic | male | 21 | 8 | 4 | 9 | 7.2 |
| | female | 25 | 13 | 4 | 10 | 9.2 |
| Other | male | 18 | 7 | 3 | 8 | 6.2 |
| | female | 16 | 9 | 5 | 3 | 6.0 |

**Table II** Ethnic-specific, age-adjusted (world population standard) incidence rates per million population for leukaemia among children (<15 years), Hawaii, 1960–64

| | *All leukaemias* | | | | *Acute lymphocytic leukaemia* | | | |
|---|---|---|---|---|---|---|---|---|
| | *Males* | | *Females* | | *Males* | | *Females* | |
| | *No.* | *Rate* | *No.* | *Rate* | *No.* | *Rate* | *No.* | *Rate* |
| White | 32 | 44.0 | 30 | 43.6 | 24 | 33.6 | 23 | 33.7 |
| Japanese | 38 | 60.9 | 18 | 34.9 | 22 | 36.6 | 13 | 26.5 |
| Filipino | 16 | 43.1 | 17 | 46.2 | 14 | 37.4 | 12 | 33.2 |
| Hawaiian | 26 | 34.0 | 33 | 47.3 | 19 | 25.2 | 14 | 20.7 |

There were few statistically significant differences between ethnic-specific incidence rates in Hawaii and rates among US whites (Table III). Leukaemia rates among Hawaii whites were similar to rates among whites in the rest of the US. The occurrence of leukaemia was more frequent among Japanese males than among US white males. Hawaiian females had a significantly higher risk of developing acute non-lymphocytic leukaemia and 'other' leukaemia than did US white females.

Time trends in the incidence of all leukaemias combined during the study period differed by ethnicity and sex (Figure 3). The incidence rate for Japanese males decreased between the time periods 1960–1972 and 1973–1984, although the difference was not statistically significant ($P=0.27$). The leukaemia rate among Hawaiian males increased non-significantly ($P=0.38$) during the study period. Generally, the range of incidence rates by ethnic group was much smaller in the latter half of the study period, varying only 13.4 million, compared with a range of incidence of 37.6 per million during the period 1960–1972.

Females in all four ethnic groups examined had increasing rates for leukaemia during the study period (Figure 3). The rate of increase was similar for all ethnic groups with the exception of whites and Japanese, although only the increase in whites was statistically significant ($P=0.05$). Unlike males, the range in age-adjusted incidence rates for females by ethnic group was rather constant during the two time periods.

### Discussion

The study of international cancer patterns is complex due to variations in case-ascertainment and reporting, disease classification, and a myriad of other possible differences. Because the Hawaii Tumor Registry is part of the SEER programme, our rates and those of the SEER whites conform to the same standards of data collection and coding. We were also able to use the disease classification scheme developed by Birch & Marsden (1987), which has been adopted as a standard by the World Health Organization.

Under-ascertainment of cases would lead to biased estimates of the true incidence rates. An attempt was made to ascertain all cancer cases in Hawaii during the period of study. We estimate that we have information on over 99% of the incident cases occurring in the state. Furthermore, autopsy and death certificate cases represent fewer than 1% of diagnoses, indicating a very high level of reporting. Since the Hawaii Tumor Registry joined the SEER programme in 1973, data collection, site, histology and ethnic classification, quality control and other aspects of case registration have been standardised. All pre-SEER data were reviewed after 1972 to ensure compatibility with SEER rules and completeness of reporting. The quality of these data are thought to be very high.

Our investigation of time trends and ethnic variation in the incidence of childhood leukaemia in Hawaii was limited by the small number of cases available for analysis. There were only 10 new cases of leukaemia reported among children each year in Hawaii and even fewer cases were available for analysis of ethnic variation in risk. Because of this limitation, incidence rates were unstable and statistical power was poor. Hence, even extreme SIRs did not attain statistical significance among ethnic minorities and the less common histological types of leukaemia.

The issue of multiple comparisons and its effect upon overall significance levels is important. As a result of the large number of comparisons ($n=20$) we made for the evaluation of ethnic-specific incidence, we expected one of the site-specific SIRs to be significant ($\alpha=0.05$) solely by chance. However, two statistically significant SIRs were observed, and an additional three were of borderline significance.

The age-adjusted and age-specific incidence for all childhood leukaemia in Hawaii was similar to that for whites in the US during the study period. Annual rates for Hawaii were 49.6 million for males and 44.8 for females. This compares to 47.8 for white males and 39.5 for white females in the combined SEER areas between 1973 and 1982 (Parkin *et al.*, 1988*b*). The slightly elevated incidence for leukaemia in Hawaii compared with all SEER areas supports findings by Breslow & Langholz (1983) for the period 1973–1977. They calculated a ratio of 1.13 for the observed rate in Hawaii to the expected rate based on age- and sex-specific leukaemia incidence for all SEER areas combined.

Pooled data from the Third National Cancer Survey and the SEER programme suggest a modest increase in the rate of acute lymphocytic leukaemia over time, particularly among females (Greenberg & Shuster, 1985). This increase was limited to the younger age groups. Our results are consistent with this finding. Although sample sizes were

**Table III** Standardised incidence ratios (SIR)[a] for leukaemia among children (<15 years) by ethnicity and sex, Hawaii, 1960–84

| | | *White* | | | *Japanese* | | | *Filipino* | | | *Hawaiian* | | | *Chinese* | | |
|---|---|---|---|---|---|---|---|---|---|---|---|---|---|---|---|---|
| *Histological type* | *Sex* | *No.* | *SIR* | *(95% CI)*[b] | *No.* | *SIR* | *(95% CI)* | *No.* | *SIR* | *(95% CI)* | *No.* | *SIR* | *(95% CI)* | No. | SIR | (95% CI) |
| All leukaemia | male | 32 | 0.9 | (0.6–1.3) | 38 | 1.4 | (1.0–1.9) | 16 | 0.9 | (0.5–1.4) | 26 | 0.7 | (0.5–1.1) | 8 | 1.3 | (0.6–2.5) |
| | female | 30 | 1.1 | (0.7–1.6) | 18 | 0.8 | (0.5–1.3) | 17 | 1.2 | (0.7–1.9) | 33 | 1.2 | (0.8–1.7) | 8 | 1.7 | (0.7–3.4) |
| Acute lymphocytic | male | 24 | 0.9 | (0.6–1.4) | 22 | 1.1 | (0.7–1.6) | 14 | 1.0 | (0.6–1.7) | 19 | 0.7 | (0.4–1.1) | 5 | 1.1 | (0.3–2.5) |
| | female | 23 | 1.1 | (0.7–1.7) | 13 | 0.8 | (0.4–1.4) | 12 | 1.1 | (0.6–1.9) | 14 | 0.7 | (0.4–1.1) | 5 | 1.4 | (0.5–3.4) |
| Acute non-lymphocytic | male | 4 | 1.0 | (0.3–2.4) | 8 | 2.2 | (1.0–4.4) | 2 | 0.9 | (0.1–3.2) | 4 | 0.9 | (0.2–2.3) | 2 | 2.5 | (0.3–9.1) |
| | female | 4 | 0.9 | (0.2–2.2) | 3 | 0.8 | (0.2–2.4) | 5 | 2.0 | (0.7–4.8) | 12 | 2.5 | (1.3–4.4) | 1 | 1.2 | (0.0–6.8) |
| Other | male | 4 | 0.9 | (0.2–2.3) | 8 | 2.3 | (1.0–4.5) | 0 | | (exp=2.3) | 3 | 0.6 | (0.1–1.9) | 1 | 1.2 | (0.0–6.9) |
| | female | 3 | 1.3 | (0.3–3.8) | 2 | 1.1 | (0.1–4.1) | 0 | | (exp=1.2) | 7 | 3.0 | (1.2–6.2) | 2 | 5.2 | (0.6–18.0) |

[a]Expected (exp) number of cases based on US white rates, Surveillance, Epidemiology and End Results data 1973–83, using scheme of Birch & Marsden (1987) for diagnostic classification; [b]95% confidence interval.

**Figure 3** Ethnic-specific, age-adjusted (world population standard) incidence rate for all leukaemias combined diagnosed among children (< 15 years) by time period at diagnosis and sex, Hawaii, 1960–84. **a**, male; **b**, female.

small, our data support a positive temporal trend in the incidence of leukaemia among females, but not males.

Incidence rates for acute non-lymphocytic leukaemia have been reported to be high among Japanese, Shanghai Chinese and New Zealand Maoris (Parkin *et al.*, 1988*b*). We found higher rates for acute non-lymphocytic and 'other' leukaemia among Japanese males and Hawaiian females compared with US whites. Correspondingly high incidence rates for two Japanese and two Polynesian groups living in different geographic areas suggests a genetic component to this disease, although the lack of similarly high rates among Hawaiian-Japanese females and Hawaiian males argues against this possibility.

Investigators have reported that the incidence of childhood leukaemia in Shanghai and Singapore Chinese was similar to that of white children in the US, Scandinavia and England, although the histological distribution was different: rates for myeloid leukaemia were higher and rates for lymphoid leukaemia were lower (Li *et al.*, 1980; Tu & Li, 1983). This observation was supported by Breslow & Langholz (1983), who compared rates for Singapore Chinese with expected rates based on data from a number of cancer registries. Parkin *et al.* (1988*a*) recently found that the ratio of acute lymphocytic leukaemia to acute non-lymphocytic leukaemia was substantially lower for Chinese than for Europeans and North American whites. These reports are generally consistent with our findings for Chinese males in Hawaii.

Incidence rates for acute lymphocytic leukaemia in white children in the US are higher than those for black children (Greenberg & Shuster, 1985; Kramer *et al.*, 1983; Parkin *et al.*, 1988*b*; Pratt *et al.*, 1988). We found that rates for acute lymphocytic leukaemia in white children were similar to the rates for this disease among Asian and Polynesian ethnic groups in Hawaii. Ethnic-specific variation in incidence was small among males. While ethnic variation was more pronounced among females, this may be attributable to more unstable rates.

The apparent increasing rate of childhood leukaemia among females in Hawaii is cause for some concern. This observation is consistent with findings from other US registries, although the increase in the rate for leukaemia elsewhere has generally been found for both sexes (Greenberg & Shuster, 1985). The ethnic-specific increase is most notable for white and Japanese females and Hawaiian males, so there are no consistent trends by ethnic group. The ethnic pattern in leukaemia incidence suggests that socioeconomic status is an unlikely factor in the aetiology of this disease, although an environmental agent(s) is likely to play some role. We will continue to monitor temporal and ethnic trends in childhood leukaemia incidence, and are currently conducting a case–control study to investigate the effects of socioeconomic status, parents' occupation and education, birth weight, maternal age, birth order and ethnicity on the development of this disease in Hawaii.

This research was supported in part by Contract N01 CN 55424 from the National Cancer Institute, US Department of Health and Human Services. The authors thank the administrators in the following hospitals for their support of this study: Kaiser Medical Center; Kuakini Medical Center; Queen's Medical Center; Straub Clinic and Hospital; St Francis Hospital; Castle Memorial Hospital; Tripler Army Medical Center; and Wahiawa General Hospital.

### References

ARMITAGE, P. (1971). *Statistical Methods in Medical Research*. John Wiley & Sons: New York.

BAILAR, J.C. & EDERER, F. (1964). Significance factors for the ratio of a poisson variable to its expectation. *Biometrics*, **20**, 639.

BIRCH, J.M. & MARSDEN, H.B. (1987). A classification scheme for childhood cancer. *Int. J. Cancer*, **40**, 620.

BRESLOW, N.E. & LANGHOLZ, B. (1983). Childhood cancer incidence: geographical and temporal variations. *Int. J. Cancer*, **32**, 703.

DUNCAN, M.H., WIGGINS, C.L., SAMET, J.M. & KEY, C.R. (1986). Childhood cancer epidemiology in New Mexico's American Indians, Hispanic Whites, and Non-Hispanic Whites, 1970–82. *J. Natl Cancer Inst.*, **76**, 1013.

GREENBERG, R.S. & SHUSTER, J.L. JR. (1985). Epidemiology of cancer in children. *Epidemiol. Rev.*, **7**, 22.

KOLONEL, L.N. (1980). Cancer patterns of four ethnic groups in Hawaii. *J. Natl Cancer Inst.*, **65**, 1127.

KRAMER, S., MEADOWS, A.T., JARRETT, P. & EVANS, A.E. (1983). Incidence of childhood cancer: experience of a decade in a population-based registry. *J. Natl Cancer Inst.*, **70**, 49.

LI, F.P., JIN, F., TU, C.-T. & GAO, Y.-T. (1980). Incidence of childhood leukemia in Shanghai. *Int. J. Cancer*, **25**, 701.

LILIENFELD, A.M. (1976). *Foundations of Epidemiology*. Oxford University Press: New York.

MUNOZ, N. (1976). Geographical distribution of pediatric tumors. *Tumori*, **62**, 145.

OYAMA, N. & JOHNSON, D.B. (1986). Hawaii Health Surveillance Program Survey Methods and Procedures. R&S Report No. 54, Hawaii State Department of Health, Research and Statistics Office.

PARKIN, D.M., STILLER, C.A., DRAPER, G.J. & BIEBER, C.A. (1988*a*). The international incidence of childhood cancer. *Int. J. Cancer*, **42**, 511.

PARKIN, D.M., STILLER, C.A., DRAPER, G.J., BIEBER, C.A., TERRACINI, B. & YOUNG, J.L. (eds.) (1988*b*). *International Incidence of Childhood Cancer*, IARC Scientific Publication 87. IARC: Lyon.

PRATT, J.A., VELEZ, R., BRENDER, J.D. & MANTON, K.G. (1988). Racial differences in acute lymphocytic leukemia mortality and incidence trends. *J. Clin. Epidemiol.*, **41**, 367.

TU, J.-T. & LI, F.P. (1983). Incidence of childhood tumors in Shanghai, 1973–77. *J. Natl Cancer Inst.*, **70**, 589.

WATERHOUSE, J., MUIR, C., SHANUGARATNAM, K. & POWELL, J. (eds.) (1982). *Cancer Incidence in Five Continents, Vol. IV*, IARC Scientific Publication 42. IARC: Lyon.

WEGNER, E.L., KOLONEL, L.N., NOMURA, A.M.Y. & LEE, J. (1982). Racial and socioeconomic status differences in survival of colorectal cancer patients in Hawaii. *Cancer*, **49**, 2208.

WORLD HEALTH ORGANIZATION (1976). *International Classification of Diseases for Oncology*. World Health Organization: Geneva.

YOUNG, J.L., PERCY, C.L. & ASIRE, A.J. (eds.) (1981). Surveillance, epidemiology and end results: incidence and mortality data: 1973–77. *Natl Cancer Inst. Monogr.*, **57**.

YOUNG, J.L., RIES, L.G., SILVERBERG, E. *et al.* (1986). Cancer incidence, survival, and mortality for children younger than age 15 years. *Cancer*, **58**, 598.